# Targeted nutritional nursing versus routine dietary care on postoperative nutritional status and recovery outcomes in patients with gastrointestinal polyps: A retrospective controlled study

**DOI:** 10.1097/MD.0000000000047545

**Published:** 2026-02-13

**Authors:** Yue Huang

**Affiliations:** a Department of Gastroenterology, Wuhan Hospital of Integrated Traditional Chinese and Western Medicine, Wuhan, Hubei, China.

**Keywords:** albumin, gastrointestinal polyps, inflammatory markers, perioperative care, postoperative recovery, targeted nutritional nursing

## Abstract

Gastrointestinal (GI) polyps are common benign lesions that may progress to malignancy if untreated. Optimizing perioperative nutritional management is crucial to enhance recovery after polypectomy. This study aimed to evaluate the effects of targeted nutritional nursing versus routine dietary care on postoperative nutritional status, inflammatory response, and recovery outcomes in patients undergoing GI polypectomy. A retrospective controlled study was conducted among 126 patients who underwent endoscopic polypectomy between January 2023 and December 2024. Patients were divided into a control group (routine dietary care) and an experimental group (targeted nutritional nursing). The targeted intervention included individualized nutrition screening (Nutritional Risk Screening 2002), personalized prescriptions (25–30 kcal/kg/d energy, 1.2–1.5 g/kg/d protein), and daily monitoring of tolerance and GI recovery. Primary outcomes included serum albumin, prealbumin, and hemoglobin. Secondary outcomes included inflammatory markers (C-reactive protein and interleukin-6), postoperative recovery parameters, and complication rates. Baseline characteristics were comparable between groups. Postoperatively, the experimental group had significantly higher albumin (40.41 ± 3.58 vs 37.23 ± 3.50 g/L, *P* < .001), prealbumin (208.89 ± 30.94 vs 192.87 ± 30.64 mg/L, *P* = .004), and hemoglobin (124.91 ± 10.74 vs 119.0 ± 10.76 g/L, *P* = .003). Inflammatory markers were significantly lower (C-reactive protein 9.32 ± 3.9 vs 13.66 ± 4.42 mg/L; interleukin-6 12.88 ± 5.67 vs 19.97 ± 7.63 pg/mL; both *P* < .001). The experimental group recovered faster with shorter anal exhaust time (1.74 ± 0.60 vs 2.18 ± 0.63 days, *P* < .001) and hospital stay (6.01 ± 1.11 vs 7.22 ± 1.81 days, *P* < .001). The total complication rate was markedly lower (12.7% vs 28.6%, *P* = .01). Targeted nutritional nursing effectively enhances postoperative nutritional and inflammatory status, accelerates GI function recovery, and reduces complication incidence in patients undergoing GI polypectomy. Incorporating individualized nutritional nursing into perioperative management is recommended to optimize surgical outcomes and recovery efficiency.

## 1. Introduction

Gastrointestinal (GI) polyps are common benign mucosal lesions originating from the epithelial or glandular tissues of the GI tract, most frequently in the stomach and colon. Although typically benign, some – especially adenomatous polyps – carry a risk of malignant transformation, underscoring the need for early detection and removal.^[1,2]^ With the advancement of minimally invasive techniques, endoscopic polypectomy have become the preferred treatments owing to their safety and faster recovery.^[3]^ Postoperative recovery depends not only on surgical technique but also on the patient’s nutritional and inflammatory status. Malnutrition, often underestimated in perioperative care, impairs immune defense, delays wound healing, and increases infection and leakage risks.^[4,5]^ Even mild deficits can disrupt protein metabolism and immune balance, prolonging hospitalization.^[6]^ Traditional postoperative dietary care mainly emphasizes the progression of food texture – from liquid to semiliquid to soft diets – without sufficient attention to nutritional composition. Such an approach neglects individual differences in energy and protein requirements, particularly in elderly or metabolically compromised patients.^[7]^ Targeted nutritional nursing offers a more personalized strategy by integrating systematic nutrition screening (e.g., Nutritional Risk Screening 2002 [NRS-2002]), individualized prescriptions, and continuous adjustment based on GI tolerance and recovery.^[8–10]^ Moreover, accumulating evidence suggests that nutritional optimization reduces inflammatory cytokine levels such as C-reactive protein (CRP) and interleukin-6 (IL-6), enhances albumin (ALB) synthesis, and accelerates the restoration of GI function.^[11,12]^ This approach aims to maintain nitrogen balance, enhance protein synthesis, and modulate inflammation, thereby accelerating recovery and reducing complications. Therefore, this retrospective controlled study aims to evaluate the effects of targeted nutritional nursing versus routine dietary care on postoperative nutritional indicators, inflammatory responses, and recovery outcomes in patients with GI polyps. The findings are expected to provide a clinical reference for optimizing perioperative nutritional nursing and improving recovery quality.

## 2. Materials and methods

### 2.1. Study design and participants

This retrospective controlled observational study was approved by the Ethics Committee of Wuhan Hospital of Integrated Traditional Chinese and Western Medicine and was conducted in accordance with the principles of the Declaration of Helsinki (Approval No: WHTCWH-2025-Clinical-018). This study was a single-center retrospective observational study conducted in accordance with the principles of the Declaration of Helsinki. A total of 158 patients diagnosed with GI polyps who were treated at our hospital between January 2023 and December 2024 were retrospectively identified through the electronic medical record system. The study was designed and reported in accordance with the Strengthening the Reporting of Observational Studies in Epidemiology guidelines. According to the predefined inclusion and exclusion criteria, 126 patients met the eligibility requirements and were included in the final analysis. Based on the type of postoperative nursing care received, the patients were divided into 2 groups: Control group (n = 63): patients who received routine dietary nursing care after GI polypectomy. Experimental group (n = 63): patients who received targeted nutritional nursing in addition to routine dietary nursing care following GI polypectomy. All patients provided informed consent for the use of their anonymized data for research purposes, and the study protocol was approved by the hospital’s Ethics Committee.

#### 2.1.1. Inclusion criteria

Patients were included in the study if they met all of the following conditions: Aged 30 to 75 years; diagnosed with GI polyps confirmed by endoscopic and histopathological examination; underwent endoscopic polypectomy in our hospital; had complete clinical and follow-up data available; were hemodynamically stable and able to tolerate postoperative oral or enteral nutrition; and provided informed consent for the use of their medical data for research purposes.

#### 2.1.2. Exclusion criteria

Patients meeting any of the following criteria were excluded: Presence of malignant GI tumors or inflammatory bowel disease; history of severe hepatic or renal dysfunction; presence of preexisting malnutrition (body mass index [BMI] < 18.5 kg/m^2^ or serum ALB < 30 g/L before surgery); severe postoperative complications, such as bleeding, perforation, or anastomotic leakage; incomplete medical records or loss to follow-up; and pregnant or lactating women.

### 2.2. Interventions

In the control group, routine dietary care was provided, consisting of gradual diet progression (liquid → semiliquid → soft) based on tolerance, basic education on eating habits, and monitoring for GI discomfort. The experimental group received targeted nutritional nursing implemented by trained nurses and dietitians, including:

① Nutritional screening and assessment using the NRS-2002 tool (weight, BMI, intake, and GI tolerance);
② Individualized nutritional prescription (25–30 kcal/kg/d energy, 1.2–1.5 g/kg/d protein, micronutrient adjustment as needed);
③ Feeding strategy prioritizing early oral or enteral nutrition with texture/timing tailored to GI function;
④ Daily monitoring of tolerance (nausea, vomiting, distension), bowel function, and glycemic control; adjusting intake plans accordingly;
⑤ Education and discharge guidance emphasizing high-protein diet, hydration, and post-discharge meal planning.

### 2.3. Outcome measures

Primary outcomes included nutritional indicators – serum ALB, prealbumin (PA), and hemoglobin (Hb) – measured within 72 hours preoperatively and on postoperative day 3 (±1 day). Secondary outcomes included inflammatory markers (CRP and IL-6), postoperative recovery indicators (time to first anal exhaust, first defecation, and length of hospital stay), and 30-day postoperative complications (nausea/vomiting, wound infection, mild ileus, diarrhea, abdominal distension, fever, hypoproteinemia, delayed wound healing, and overall incidence).

### 2.4. Data collection and quality control

Two independent investigators extracted demographic, clinical, and surgical data using a standardized template. Discrepancies were resolved by discussion with a third investigator to ensure data accuracy and completeness.

### 2.5. Statistical analysis

Data normality was tested using the Shapiro–Wilk test. Normally distributed variables were expressed as mean ± standard deviation and compared between groups using independent-samples *t*-tests. Non-normally distributed data were analyzed with Mann–Whitney *U* tests. Within-group comparisons were made using paired *t*-tests. Categorical variables were expressed as counts and percentages and compared using χ^2^ or Fisher exact tests. Two-sided *P* < .05 was considered statistically significant. Statistical analyses were performed using Statistical Package for the Social Sciences version 26.0 (International Business Machines Corporation [IBM], Armonk) or R version 4.3 (The R Foundation for Statistical Computing, Vienna, Austria).

### 2.6. Missing data and sensitivity analysis

Cases with missing primary outcome data were excluded from that analysis. No imputation was performed. Sensitivity analyses were conducted using nonparametric tests when normality assumptions were violated to ensure robustness of results.

## 3. Results

### 3.1. Comparison of baseline characteristics between the 2 groups

A total of 126 patients were enrolled, including 63 in the control group and 63 in the experimental group. No significant differences were found between the 2 groups in demographic or clinical characteristics (all *P* > .05). The gender distribution (male/female: 37/26 vs 39/24), mean age (55.18 ± 19.6 vs 55.91 ± 20.1 years), and BMI (23.48 ± 2.52 vs 24.27 ± 2.38 kg/m^2^) were comparable. Lesion sites were mainly gastric or colorectal (29/34 vs 33/30). Multiple polyps were more common in both groups (10/53 vs 13/50). Lifestyle and comorbidity factors, including smoking, alcohol use, diabetes, hypertension, and education level, showed no statistical difference (Table 1).

**Table 1:** Baseline characteristics of patients.

Variable	Control (n = 63)	Experimental (n = 63)	*P*-value
Male/Female (n)	37/26	39/24	.81
Age (yr)	55.18 ± 19.6	55.91 ± 20.1	.69
BMI (kg/m^2^)	23.48 ± 2.52	24.27 ± 2.38	.06
Lesion site (gastric/colorectal)	29/34	33/30	.58
Polyp number (single/multiple)	10/53	13/50	.49
Smoking history [n (%)]	28 (44.4%)	30 (47.6%)	.71
Alcohol consumption [n (%)]	19 (30.2%)	15 (23.8%)	.42
Diabetes mellitus [n (%)]	10 (15.9%)	7 (11.1%)	.46
Hypertension [n (%)]	15 (23.8%)	20 (31.7%)	.32
Education level (≤High school/≥College)	39/24	35/28	.37

BMI = body mass index.

### 3.2. Effect of targeted nutritional nursing on postoperative nutritional status

Before surgery, there were no statistically significant differences between the control and experimental groups in serum ALB, PA, or Hb levels (all *P* > .05), indicating comparable baseline nutritional status. Specifically, the mean preoperative ALB was 34.92 ± 3.49 g/L in the control group and 36.04 ± 3.58 g/L in the experimental group (*t* = 1.79, *P* = .077). Similarly, preoperative PA and Hb levels showed no significant differences between groups (PA: *t* = 0.43, *P* = .670; Hb: *t* = 1.84, *P* = .068). After the intervention, the experimental group demonstrated significantly higher nutritional parameters compared with the control group. Postoperative ALB increased to 40.41 ± 3.58 g/L in the experimental group versus 37.23 ± 3.50 g/L in the control group (*t* = 5.04, *P* < .001). Likewise, PA levels were significantly elevated in the experimental group (208.89 ± 30.94 mg/L) compared with the control group (192.87 ± 30.64 mg/L, *t* = 2.92, *P* = .004). Hb levels also showed a notable postoperative improvement in the experimental group (124.91 ± 10.74 g/L) relative to the control group (119.0 ± 10.76 g/L, *t* = 3.08, *P* = .003; Table 2).

**Table 2:** Between-group comparison of nutritional indicators.

Indicator	Time	Control (Mean ± SD)	Experimental (Mean ± SD)	*t*-Value	*P*-value
ALB	Before	34.92 ± 3.49	36.04 ± 3.58	1.79	.077
ALB	After	37.23 ± 3.5	40.41 ± 3.58	5.04	<.001
PA	Before	181.74 ± 24.82	183.46 ± 19.84	0.43	.670
PA	After	192.87 ± 30.64	208.89 ± 30.94	2.92	.004
Hb	Before	115.23 ± 8.4	118.34 ± 10.45	1.84	.068
Hb	After	119.0 ± 10.76	124.91 ± 10.74	3.08	.003

ALB = albumin, Hb = hemoglobin, PA = prealbumin, SD = standard deviation.

### 3.3. Effect of targeted nutritional nursing on postoperative inflammatory markers

Before the intervention, no statistically significant differences were observed in CRP or IL-6 levels between the control and experimental groups (CRP: *P* = .73; IL-6: *P* = .66), indicating that both cohorts were comparable with respect to baseline inflammatory status. Postoperatively, both inflammatory parameters declined in each group; however, the reduction was significantly more pronounced among patients who received targeted nutritional nursing. Specifically, postoperative CRP levels were markedly lower in the experimental group (9.1 ± 3.7 mg/L) compared with the control group (13.5 ± 4.8 mg/L, *t* = 5.13, *P* < .001). Likewise, IL-6 concentrations demonstrated a substantial decrease, reaching 12.88 ± 5.67 pg/mL in the experimental group versus 19.97 ± 7.63 pg/mL in the control group (*t* = 5.40, *P* < .001; Table 3).

**Table 3:** Comparison of postoperative serum inflammatory markers between groups.

Indicator	Time	Control (Mean ± SD)	Experimental (Mean ± SD)	*t*-Value	*P*-value
CRP	Before	18.58 ± 5.73	20.49 ± 5.86	1.86	.066
CRP	After	13.66 ± 4.42	9.32 ± 3.9	5.84	<.001
IL-6	Before	26.84 ± 8.17	27.62 ± 6.24	0.6	.551
IL-6	After	19.97 ± 7.63	12.88 ± 5.67	5.91	<.001

CRP = C-reactive protein, IL-6 = interleukin-6, SD = standard deviation.

### 3.4. Effect of targeted nutritional nursing on postoperative recovery

Postoperative recovery was faster in the experimental group across all measured indicators. The time to first anal exhaust was significantly shorter in the experimental group (1.74 ± 0.60 days) than in the control group (2.18 ± 0.63 days, *t* = 4.02, *P* < .001). Similarly, the time to resume diet was reduced in the experimental group (2.85 ± 0.95 days) compared with the control group (3.64 ± 1.01 days, *t* = 4.48, *P* < .001).

The length of hospital stay was also significantly shorter in the experimental group (6.01 ± 1.11 days) than in the control group (7.22 ± 1.81 days, *t* = 4.52, *P* < .001; Table 4).

**Table 4:** Presents the comparison of postoperative recovery parameters between the control and experimental groups.

Indicator	Control (Mean ± SD)	Experimental (Mean ± SD)	*t*-Value	*P*-value
Exhaust time	2.18 ± 0.63	1.74 ± 0.6	4.02	<.001
Resume diet	3.64 ± 1.01	2.85 ± 0.95	4.48	<.001
Hospital stay	7.22 ± 1.81	6.01 ± 1.11	4.52	<.001

SD = standard deviation.

### 3.5. Effect of targeted nutritional nursing on postoperative

Complications The overall complication rate was lower in the experimental group than in the control group (12.7% vs 28.6%, *P* = .01). Among specific complications, nausea and vomiting were observed in 6 patients (9.5%) in the control group and 3 patients (4.8%) in the experimental group (*P* = .29). Wound infection occurred in 3 patients (4.8%) in the control group and 2 patients (3.2%) in the experimental group, with no statistically significant difference (*P* = .48).

Mild ileus occurred in 3 patients (4.8%) in the control group and 1 patient (1.6%) in the experimental group (*P* = .03). Fever was recorded in 4 patients (6.3%) in the control group and 1 patient (1.6%) in the experimental group (*P* = .04). Other mild complications, including diarrhea, abdominal distension, hypoproteinemia, and delayed wound healing, showed no significant intergroup differences (*P* > .05). No severe postoperative complications (Table 5).

**Table 5:** Comparison of postoperative complications between groups.

Complication	Control (n = 63)	Experimental (n = 63)	χ^2^/Fisher exact test	*P*-value
Nausea/vomiting	6 (9.5%)	3 (4.8%)	1.12	.29
Wound infection	3 (4.8%)	2 (3.2%)	0.50	.48
Mild ileus	3 (4.8%)	1 (1.6%)	4.58	.03*
Diarrhea	4 (6.3%)	2 (3.2%)	1.85	.17
Abdominal distension	5 (7.9%)	2 (3.2%)	3.42	.06
Fever	4 (6.3%)	1 (1.6%)	4.17	.04*
Hypoproteinemia	3 (4.8%)	1 (1.6%)	2.88	.09
Delayed wound healing	2 (3.2%)	0 (0.0%)	3.10	.08
Total incidence	18 (28.6%)	8 (12.7%)	6.23	.01*

* indicated *P* < .05.

## 4. Discussion

This retrospective study demonstrated that targeted nutritional nursing significantly improved postoperative nutritional status, attenuated inflammatory responses, and promoted faster recovery in patients undergoing GI polypectomy compared with routine dietary care. The individualized approach led to higher postoperative serum ALB, PA, and Hb levels, lower CRP and IL-6 concentrations, faster bowel function recovery, and fewer postoperative complications. These findings highlight the clinical value of incorporating targeted nutritional strategies into perioperative management for patients with GI polyps.

Malnutrition remains a prevalent yet frequently overlooked risk factor among gastroenterology patients, which is particularly pronounced in those undergoing GI surgery.^[13,14]^ Nutritional deficits can impair immune competence, delay wound healing, and increase susceptibility to infection and other complications. Routine dietary care, although widely practiced, mainly focuses on dietary progression and tolerance, with limited attention to nutrient composition or individual energy requirements.^[15]^ Consequently, many patients fail to achieve adequate protein and caloric intake during the critical early recovery period. In contrast, targeted nutritional nursing integrates structured nutritional screening (e.g., NRS-2002), individualized prescription, and continuous monitoring of tolerance and metabolic status. This proactive approach allows dynamic adjustment of feeding routes and nutrient proportions according to the patient’s physiological condition. Similar to findings from previous clinical trials,^[16,17]^ our results suggest that personalized nutritional management helps preserve nitrogen balance, stimulates protein synthesis, and maintains plasma protein levels, which collectively enhance postoperative recovery. The observed postoperative improvement in ALB and PA supports the role of individualized care in promoting anabolic recovery and restoring nutritional reserves.

Inflammation is another key determinant of surgical recovery, elevated levels of pro-inflammatory cytokines such as CRP and IL-6 are commonly observed after GI surgery and are closely associated with delayed healing and postoperative morbidity.^[18,19]^ In this study, targeted nutritional nursing markedly reduced CRP and IL-6 levels postoperatively, indicating an attenuation of the systemic inflammatory response. This effect may be attributed to early initiation of enteral or oral feeding, which preserves gut mucosal integrity, minimizes bacterial translocation, and regulates immune activation.^[20,21]^ Early nutrition also helps maintain intestinal motility and barrier function, reducing the risk of postoperative ileus and infectious complications. Furthermore, patients in the experimental group demonstrated faster GI recovery – reflected by earlier anal exhaust, defecation, and bowel sound recovery – as well as shorter hospitalization. These improvements are consistent with literature reporting that optimized perioperative nutrition accelerates GI motility and functional recovery.^[22,23]^ By improving tissue oxygenation and reducing inflammatory stress, targeted nutrition facilitates earlier mobilization and discharge.

Notably, no increase in adverse events was observed, supporting the safety and feasibility of implementing this intervention in clinical settings. Postoperative complications significantly affect patient prognosis and healthcare burden. In the present study, the total complication rate in the experimental group was almost half that of the control group, mainly due to reductions in nausea, vomiting, ileus, and infection. Previous studies have similarly demonstrated that perioperative nutritional support lowers the risk of infection and metabolic complications by stabilizing immune function and improving microcirculation.^[24,25]^ The absence of severe adverse events further supports that targeted nutrition does not increase the risk of postoperative complications and may improve resilience to physiological stress.

The findings of this study have important clinical implications. From a nursing perspective, targeted nutritional management provides a patient-centered framework that emphasizes assessment, intervention, and education. By integrating dietitians and specialized nurses into perioperative care, the intervention enhances interdisciplinary collaboration and ensures consistent nutritional monitoring. Moreover, the approach is cost-effective, requiring no specialized equipment beyond structured assessment and tailored feeding guidance, making it feasible for implementation in general hospitals.

Several limitations of this study should be acknowledged. First, due to the retrospective observational design, potential confounding factors could not be fully controlled. Variables such as polypectomy technique (including HSP, cold snare polypectomy, and endoscopic mucosal resection), polyp size, intraoperative blood loss, and the severity of underlying comorbidities were not adjusted using multivariate models or propensity score matching. These factors may have influenced postoperative nutritional status, inflammatory response, and recovery outcomes. Although baseline characteristics were comparable between groups, residual confounding cannot be completely excluded. Therefore, the observed associations should be interpreted with caution. Future prospective, multicenter randomized controlled trials with comprehensive adjustment for surgical and clinical variables are warranted to further validate the effectiveness of targeted nutritional nursing.

In conclusion, this study reinforces that perioperative nutrition is not merely an adjunct but an integral component of therapy, pivotal to enhancing recovery after GI polypectomy. The systematic integration of targeted nutritional care into standard perioperative management optimizes metabolic stability, mitigates inflammatory responses, and improves clinical outcomes. As evidence continues to accumulate, establishing standardized nutritional protocols will be instrumental in advancing enhanced recovery programs and elevating overall healthcare quality.

## 5. Conclusion

Targeted nutritional nursing, when applied as a structured and individualized component of perioperative care, significantly improves postoperative nutritional status, reduces inflammatory responses, and enhances recovery in patients undergoing GI polypectomy. Compared with routine dietary care, this approach leads to faster GI recovery, shorter hospital stays, and fewer postoperative complications. Integrating targeted nutritional nursing into standard perioperative care is therefore recommended to improve patient outcomes and optimize clinical recovery pathways.

## Author contributions

**Conceptualization:** Yue Huang.

**Data curation:** Yue Huang.

**Formal analysis:** Yue Huang.

**Funding acquisition:** Yue Huang.

**Investigation:** Yue Huang.

**Writing – original draft:** Yue Huang.

**Writing – review & editing:** Yue Huang.
